# Perception of Ghanaian Primigravidas Undergoing Their First Antenatal Ultrasonography in Cape Coast

**DOI:** 10.1155/2020/4589120

**Published:** 2020-10-23

**Authors:** Emmanuel Kobina Mesi Edzie, Klenam Dzefi-Tettey, Philip Narteh Gorleku, James William Ampofo, Albert Dayor Piersson, Abdul Raman Asemah, Henry Kusodzi, Richard Ato Edzie

**Affiliations:** ^1^Department of Medical Imaging, School of Medical Sciences, College of Health and Allied Sciences, University of Cape Coast, P.M.B. University of Cape Coast, Cape Coast, Ghana; ^2^Department of Radiology, Korle Bu Teaching Hospital, P.O. Box KB77, Korle Bu, Accra, Ghana; ^3^Department of Imaging Technology and Sonography, School of Allied Health Sciences, College of Health and Allied Sciences, University of Cape Coast, P.M.B. University of Cape Coast, Cape Coast, Ghana

## Abstract

Ultrasound scans have become an essential requirement of pregnancy care in countries with developed health services and increasingly being used in medical practice in Ghana as well. The aim of this study was to find out the perception of primigravidas experiencing antenatal ultrasonography for the first time in Cape Coast. This was a descriptive, prospective study which employed the use of a questionnaire to obtain data from 384 consented respondents, who were primigravidas experiencing antenatal ultrasonography for the first time in three selected public health facilities in Cape Coast Metropolis over a six-month period. Sociodemographic data, reasons for undergoing antenatal ultrasound, their expectations, knowledge in fetal abnormalities, and suggestions to help improve their future experiences were collected. The data were analyzed using SPSS software, version 20.0 (SPSS Inc., Chicago, IL, USA). Out of a total number of 384 respondents, 87.8% of them knew about what ultrasound is used for. 87.5% scanned because a doctor or midwife requested for the scan whilst 53.9% scanned to check for fetal abnormalities. 98.4% indicated that ultrasound scanning has positive effects on pregnancy outcome. An expensive service was stated as a negative reason that would influence the decision to undergo the examination next time; nonetheless, 90.4% would recommend it to other women and suggested showing the fetus on monitor while scanning and providing accurate findings would make their future experiences better. The perception of the primigravidas was largely positive. Checking for fetal abnormalities was a major reason for the scans, although their knowledge in specific fetal abnormalities was low. They expected to know the fetal sex, but that was not a major reason for scanning. Showing them the monitor was the most frequent suggestion to make future experience better.

## 1. Introduction

Ultrasound technology, initially developed in the advanced world, is now continuously evolving in other parts of the world and has become a crucial part of antenatal care in developing countries [1–4]. Today, the use of ultrasound has become a common practice for routine antenatal care in health centres; consequently, there has been a lot of interventional packages like specialized ultrasound training to improve women's experience with antenatal ultrasonography [5]. Its use has shown benefits for women who perceive antenatal scanning as a means for reassurance about the health and wellbeing of their fetus, while for others, ultrasound might be injurious and can harm or deform the fetus [6, 7]. The importance of ultrasound scanning in prenatal care cannot be overemphasized although there may be variations in the degree of diagnostic accuracy of ultrasound depending on when pregnant women present themselves for an ultrasound examination (in the estimation of gestational age, determination of fetal sex and fetal anomaly, etc.) [8] which can give adequate and timely information for the necessary decisions to be made and also helps in keeping pregnant women informed about the wellbeing of their unborn babies and keeping them reassured [6, 7]. A correct determination of gestational age is needed to distinguish preterm newborns from newborns who are with low birth weight (but not preterm), which is important because the needed interventions may differ [9]. The regular use of ultrasound scan in the management of pregnancy has dramatically improved the detection of high-risk pregnancies and increased prenatal care attendance although there is no consensus about whether the use of obstetric ultrasound has the ability to decrease maternal and child mortality in low- and middle-income countries [10–12].

Across many countries, women have rated ultrasonography as one of the most crucial part of their antenatal care [13]. In view of the overwhelming benefits of ultrasound in women, many women in Africa have not fully taken advantage of this imaging modality partly due to the misconceptions they have or have heard about the procedure elsewhere [14]. In sub-Saharan Africa, the kind of perception women have on antenatal ultrasound varies and greatly depends on the educational status attained [15, 16]. In some African societies, there are some cultural resistances to ultrasound usage in obstetrics, where it runs up against traditional myths and taboos. Some ethnic groups consider it a bad luck to reveal the content of the uterus in pregnancy and to be able to see the fetus [17, 18]. However, in some parts of sub-Saharan Africa, women have positive views about ultrasound services and considered ultrasound as a tool that could make pregnancy and childbirth safer [19]. For instance, in the rural Botswana district hospital, pregnant women showed signs of trusting the ultrasound results more than their own bodily sensations to confirm a live fetus [20].

In Ghana, there are many public and private ultrasound-imaging centres, and antenatal care practitioners are equally increasingly referring their clients for ultrasound evaluation of their pregnancies [21]. Many pregnant women undergo antenatal ultrasound imaging examination daily in Cape Coast and it will be interesting to know how these pregnant women perceive antenatal ultrasonography especially for first-time users. The perception of primigravidas about antenatal ultrasonography has been scarcely described in this locality. Therefore, this study is designed to determine the perception of the primigravidas without prior experience of antenatal ultrasonography scan in Cape Coast.

## 2. Materials and Methods

This was a cross-sectional prospective study conducted from 1st May to 30th September 2019 in the Cape Coast Metropolis of Ghana using a questionnaire. The questionnaire was designed to address how the primigravidas experiencing their first antenatal ultrasound perceive the use of ultrasonography, with regard to their knowledge and expectations just before their first antenatal ultrasound examination and their suggestions and recommendations just after undergoing the examination. This was done in order to capture the perception of respondents on each variable under consideration.

The respondents were primigravidas experiencing their first antenatal ultrasonography from the Cape Coast Teaching Hospital (CCTH), Adisadel Clinic, and Ankaful General Hospital (AGH) and those who had experienced antenatal ultrasonography before were excluded. CCTH is located in the central part of the Cape Coast Metropolis of the Central Region of Ghana and is the largest public health institution in the region, which offers tertiary services. It also serves as a referral point for both public and private primary and secondary health facilities in the Central Region. Adisadel Clinic and Ankaful General Hospital, both being secondary health facilities, are located in the southern part and the north-western part of Cape Coast Metropolis, respectively. These selected sites are the main public health facilities that offer ultrasound services in Cape Coast Metropolis.

The sample size of 384 was obtained using the population of women in the Central Region of Ghana from the National Census 2010, which was estimated to be about 1,151,751 (2010 Population and Housing Census) [22] and using the statistical software sample size calculator [23] at a confidence level of 95% with an error margin of 5%. The respondents were obtained from outpatients visiting these facilities for antenatal ultrasound imaging. Simple random sampling technique was used to select respondents for the study in each facility after proportionately allocating the number of respondents that were selected from each facility based on how busy the ultrasonography unit was in the past year. CCTH had an annual ultrasound imaging examinations of 8436 for 2018, Adisadel Clinic had 3120, and AGH had 2080 for 2018 from the primary records of the respective facilities giving a total of 13,636 examinations. Using simple proportions, 237, 88, and 59 respondents were obtained from CCTH, Adisadel Clinic, and Ankaful General Hospital, respectively, as shown in Table 1.

Between the hours of 7:00 AM and 8:00 AM each day, pregnant women who reported to the ultrasound unit were approached and those who were primigravidas irrespective of their gestational age and were there for their first scan were identified. The nature of the study was explained to them and all of those willing to participate were recruited and given consent form to sign. Numbers were assigned to these consented participants and a maximum of five were picked through balloting every day. The questionnaires were pretested to check for clarity, reliability, and validity. The pretested questionnaires with both open- and close-ended questions were administered to these five randomly selected participants until the target for each site was attained. The questionnaires were administered in two parts, the first part, which featured questions to elicit their knowledge and views about the procedure, their expectations during the procedure, and the reason why they were there for the ultrasound examinations, before entering the scan room and the second part, which included questions like whether participants would recommend antenatal ultrasound scan to other women and why, and their suggestions to the doctors to make their next experience better, after experiencing the scan.

The questionnaires were retrieved and carefully checked for completeness. The data obtained from both open- and close-ended questions were grouped, organized, coded, and inputted into Excel (2010) and analyzed using SPSS software, version 20.0 (SPSS Inc., Chicago, IL, USA) for Windows to obtain frequencies, charts, and percentages. A Chi-squared test for independence was conducted to establish the association between the levels of education of the participants and various responses regarding their knowledge on congenital abnormalities and reasons for antenatal ultrasonography. The level of significance was specified at *P* < 0.05.

### 2.1. Ethical Consideration

Ethical clearance for this study was obtained from the Institutional Review Board of the CCTH. Written informed consent was obtained from each participant before the questionnaire was administered. Those who did not consent were excluded. Confidentiality and anonymity were ensured. The questionnaires were safely stored in a locked cabinet at the Department of Radiology of CCTH. This study conformed to the 1975 Declaration of Helsinki.

## 3. Results

### 3.1. Sociodemographic Characteristics of Participants

A total of 384 primigravidas attending antenatal ultrasound imaging in Cape Coast met the inclusion criteria and were recruited into the study. The mean age of the primigravidas was 29.1 years (SD 1.14 years). Sociodemographic characteristics (age ranges, educational status, religion, occupation, and marital status) are shown in Table 2.

The majority of the respondents 337 (87.8%) indicated that they knew what ultrasound is used for. Almost all the women 378 (98.4%) indicated that ultrasound has positive effects on pregnancy outcome (generally safe, check fetal wellbeing, position and presentation, etc.) with just few 6 (1.6%) stating ultrasound has negative effects on pregnancy outcome (time wasting, the wrong diagnosis of fetal sex and abnormality, the wrong estimation of date of delivery, etc.). The decision of the women to undergo their next ultrasound scan would be influenced by the cost of the ultrasound scan in 374 (97.4%) of responses and stated the following as reasons: expensive service in 171 (45.7%), free service in 119 (31.8%), and service captured under NHIS in 84 (22.5%) responses. The cost was not a factor for 10 (2.6%) of the respondents (Table 3).

### 3.2. The Women's Views on Frequency of Ultrasound Imaging during the Pregnancy

The majority of the respondents 133 (34.6%) indicated that they wanted antenatal ultrasound imaging done three times in a pregnancy. 93 (24.2%), 70 (18.2%), and 48 (12.5%) of the respondents wanted ultrasound scan performed for them two times, more than three times, and once, respectively. Out of the 384 respondents, 40 (10.4%) did not know the number of times they wanted ultrasound imaging done during a pregnancy.

The commonest reason for women to undergo ultrasound imaging was because a doctor or midwife requested it in 336 (87.5%) of the respondents. 184 (47.9%) had the scan done because they thought it was relevant to the management of their pregnancy, but 200 (52.1%) did not think so. Only 146 (38.0%) of the 384 women would do an ultrasound imaging to check the gender of the fetus. Out of the total of 384 women, 207 (53.9%) of the women were interested in checking for abnormalities in the fetus, 150 (39.1%) wanted to confirm the estimated date of delivery, and 133 (34.6%) were interested in checking for the number of fetuses. None of the pregnant women undertook the scan because religious leaders advised them to do so. Regarding expectations of the participants for antenatal ultrasound scan, responses from the participants indicated that 223 (58.1%) expected to know pregnancy-related complications, 224 (58.3%) wanted to know the gender of the fetus, while 221 (57.6%) expected to be informed on any abnormalities in the fetus during their scan. However, 184 (47.9%) expected to be informed on whether the placenta is located normally, 141 (36.7%) wanted to be informed on the number of fetuses they were carrying, and 126 (32.8%) indicated that they wanted to see their fetus before birth (Table 4).

**Figure 1:**
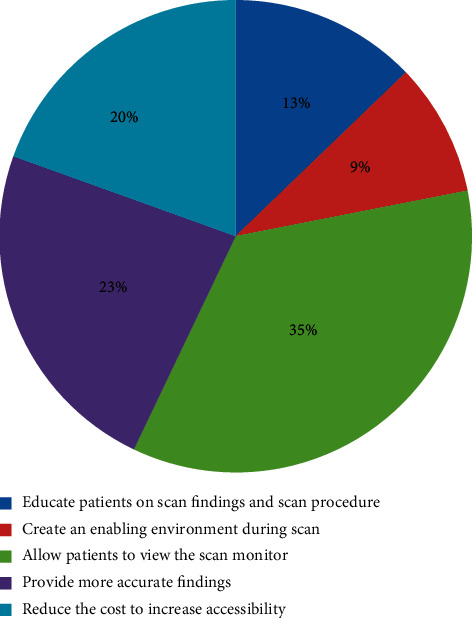
Participants' suggestions to doctors to make their experience better during their next ultrasound imaging.

The majority of the respondents 135 (35.2%) suggested that they should be allowed to view the scan monitor during the scanning process; the other suggestions are shown in Figure 1.

**Figure 2:**
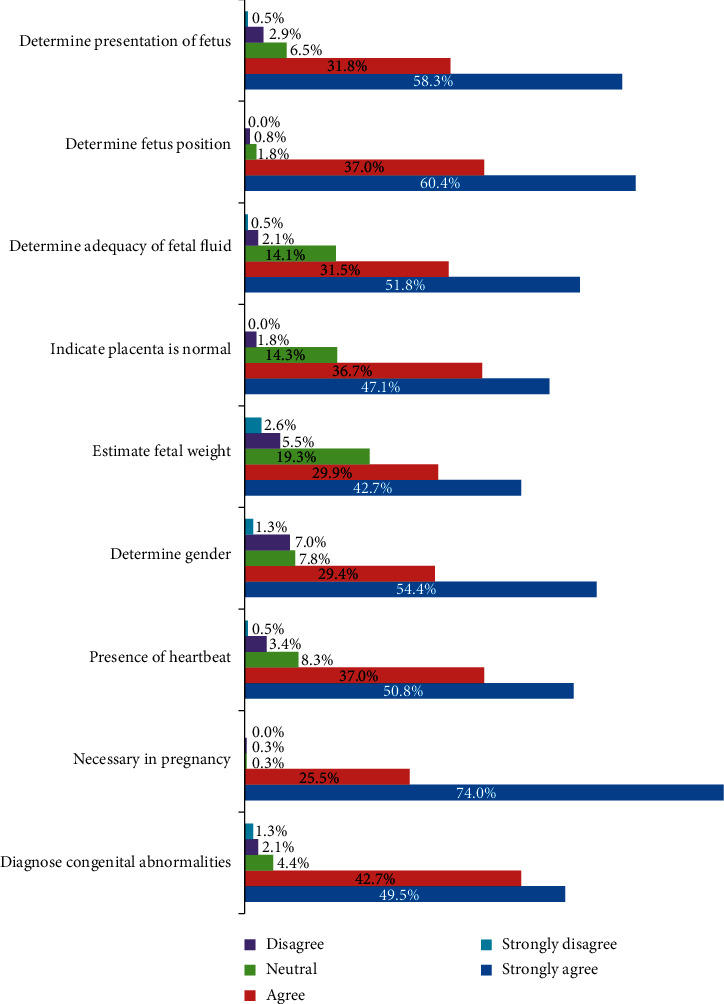
Knowledge of respondents about the ultrasound imaging.

The degree to which respondents agreed or disagreed with the benefits of antenatal ultrasound imaging showed that the majority 354 (92.2%) of the participants, comprising 190 (49.5%) strongly agreeing and 164 (42.7%) agreeing that ultrasound can help in diagnosing some congenital abnormalities, while only 13 (3.4%) strongly or just disagreed that ultrasound can help in diagnosing some congenital abnormalities. 17 (4.4%) were neutral. Almost all the respondents 382 (99.5%) further affirmed that ultrasound imaging is necessary in pregnancy comprising 284 (74.0%) and 98 (25.5%) strongly agreeing and just agreeing, respectively. None strongly disagreed that ultrasound was necessary in pregnancy with only 1 (0.3%) just disagreeing, and 1 (0.3%) was neutral. Also 209 (54.4%) and 113 (29.4%) strongly agreed and agreed, respectively, that ultrasound can help determine the gender of the fetus with a composite 322 (83.8%) agreeing, whereas 30 (7.8%) were neutral and 27 (7.0%) and 5 (1.3%) disagreed and strongly disagreed, respectively. 374 (97.4%) of the respondents indicated that they strongly agreed and agreed that ultrasound can determine the position of the fetus; 3 (0.8%) disagreed and 7 (1.8%) were neutral. With respect to the ability of antenatal ultrasound imaging to determine the presentation of the fetus, a majority of the women 336 (90.1%), comprising 224 (58.3%) who strongly agreed and 112 (31.8%) agreed, 25 (6.5%) were neutral, 11 (2.9%) disagreed, and 2 (0.5%) strongly disagreed as shown in Figure 2.

**Figure 3:**
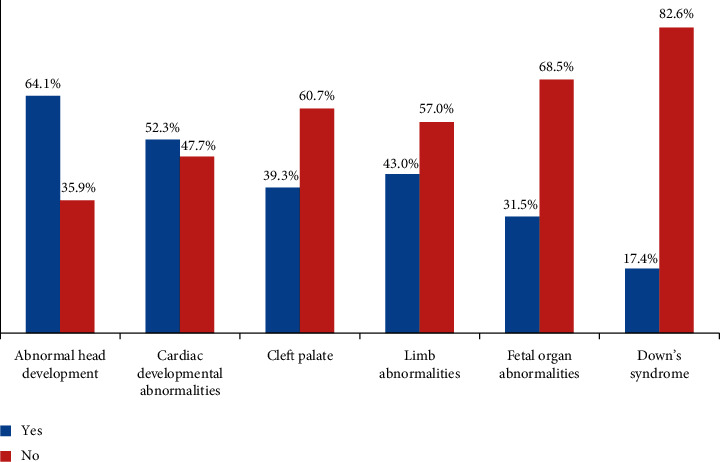
Knowledge of congenital abnormalities that ultrasound can diagnose.

Out of the total number of 384 respondents, 246 (64.1%) agreed that ultrasound can diagnose abnormal head development and the rest did not. 201 (52.3%) also indicated that it could help diagnose cardiac developmental abnormalities whereas 151 (39.3%) stated that it can diagnose cleft palate. The responses further showed that 165 (43.0%), 121 (31.5%), and 67 (17.4%) of the respondents specified that ultrasound could help diagnose limb abnormalities, fetal organ abnormalities, and Down's syndrome, respectively (Figure 3).

A high number, 347 (90.4%), of the participants would recommend antenatal ultrasound imaging to other women; the rest 37 (9.6%) would not. The majority of the 347 respondents, 107 (30.8%), cited that their main reasons for recommending the antenatal ultrasonography were to ensure the detection of the fetal position, development, and pregnancy-related complications. Detection of congenital abnormalities was also indicated by 97 (28.0%) respondents as another reason for their recommendation of ultrasound scan followed by 86 (24.8%) that stated the management of pregnancy and planning for recommending ultrasonography scan. It was also stated by 57 (16.4%) of the respondents that knowing the estimated date of delivery and confirming pregnancy was also a reason for recommending the scan (Table 5).

## 4. Discussion

Since its perfected clinical use in the 1950s, ultrasound plays an essential role in antenatal care around the world [24]. The modal age range for this study was 21–30 years. This agrees with the same age range reported by Saleh et al. [15]. The mean age for the participants was 29.1 (SD 1.14) years, which is similar to the 29.4 years reported in Sweden by Georgsson Öhman and Waldenström [25] and deviated from the 27.7 years reported in Nottingham by Whynes [26] and 28.1 years in Ghana by Mensah et al. [21]. The majority of the primigravidas (76.3%) in the study were married, which is expected in our setting where most gravid women are usually married corroborated by Mensah et al. [21] and Adekanmi et al. [6].

The majority of the participants (98.4%) stated that ultrasound has mainly positive effects on pregnancy outcome (generally safe, check fetal wellbeing, position and presentation, etc.). Saleh et al. [15] reported that the ultrasound procedure is safe, which is one of the positive effects found in this study. This finding is encouraging, as it will not deter them from undergoing the examination. The decision of the respondents to undergo the scan the next time would be influenced by the cost of the examination in 97.4% of cases and most of them stated expensive cost as their main reason in this study. Most of the participants (34.6%) indicated that the scans should be done three times during the course of a pregnancy contrary to what was found in Nigeria by Saleh et al. [15] that showed that most (41.0%) wanted it twice. This study found that the modal number of scans the women wanted to undergo was 3.0 which is higher than the 2.0 reported in Ghana by Mensah et al. [21] and in UK by Whynes [26]. These differences may be due to the fact that we looked at the expected number of scans per pregnancy while the studies done in Ghana and UK above looked at the actual number of scans that were done.

The main reasons why the respondents had their antenatal scans were that a doctor or midwife requested for it in 87.5% of responses. This agrees with a study conducted by Mensah et al. [21] which reported that 100% of the requests were from the doctor or midwife. This may be explained by the fact that in Ghana, requests are made by practitioners for antenatal ultrasonography during routine antenatal care in order to monitor the progress of pregnancy. Irrespective of their educational background (Table 6), the majority of the participants from this study (53.9%) wanted to know if there were any fetal abnormalities (Table 4). This is similar to a study conducted in Sweden, which also reported the majority of respondents who wanted confirmation of normality [25]. The knowledge about the normality status of the fetus turns to reassure pregnant women about the health and safety of the fetus.

39.1%, 38.0%, and 34.6% indicated they wanted to know their estimated date of delivery, gender of the fetus, and the number of the fetuses, respectively. These may be low due to the fact that those variables are out of control of the women and therefore were not the main reasons they considered for undergoing the scans. Nonetheless, a higher percentage (83.8%) of the women knew the capability of ultrasound in detecting the gender of the fetus even though it was not a major reason for the scan. A reason that may account for this disparity may be sociocultural, since inheritance and lineage may be maternal or paternal in various parts of the country and also, Ghanaian custom views children as members of either their mother's or father's lineage (extended family), but not both. The patrilineal custom charges a man's lineage with caring for his widow and children, while matrilineal custom places this burden on the widows' lineage—her father, brother, and uncles—and hence there is no emphasis placed on gender [27].

Religious considerations did not influence the participants in their decision to undergo the scanning, though all the respondents were either Christians or Muslims. This is interesting to note because Ghanaian societies are highly influenced by religion in almost all spheres of life, which corroborates with a study in Ghana, which reported prayers and revelation, reversing negative dreams, laying of hands, and anointing pregnant women as pastor's spiritual interventions in pregnancies by Aziato et al. [28].

It was noted that even before undergoing ultrasound scan for the first time, these primigravidas had a wide range of expectations for the ultrasound examination including knowing gender of the fetus (58.3%), fetal abnormalities (57.6%), seeing of the fetus before birth (32.8%), knowing the number of fetuses (36.7%), etc. as shown in Table 4 which were much lower than that of Irish counterparts (90%, 85%, 62%, and 99%, respectively), as reported by [29]. The higher degree of expectation of the Irish respondents may be because all the participants were literates whilst in our study the literacy level of most of the respondents was low.

With regards to the knowledge of the respondents, 87.8% of the respondents had knowledge about what ultrasound is used for, similar to what was found in Nigeria by Agbata et al. [30] who reported that a high percentage of respondents had knowledge on the use of ultrasound in pregnancy. This is consistent with the report showing that knowledge on ultrasound is high, since there is an extensive use of ultrasound in health care today as part of routine antenatal care as well as unlimited access to information, according to Saleh et al. [15]. The majority (above 80%) agreed that ultrasound can determine the presentation of the fetus, the presence of a heartbeat, indicate whether the placenta is normal, congenital abnormalities, the fetal gender, the position of the fetus, estimate fetal weight, adequacy of fluid around the fetus, and is necessary in pregnancy (Figure 2). This is comparable to the findings with some similar parameters as reported by [29]. This confirms that the women exhibited a high degree of knowledge about obstetric ultrasonography, which is laudable. Our study found that the expressed knowledge of the participants on specific congenital and chromosomal abnormalities was varied, but generally low (Figure 2), even though we found a significant association between their level of education and knowledge on some specific congenital abnormalities like cleft palates, fetal organ abnormalities, and Down's syndrome. This result is comparable to the varied results reported in Ireland with regards to fetal malformations and chromosomal abnormalities [29]. This may be due to the varying depth of technical knowledge.

The percentages of suggestions made by our respondents to make their next experience better in this study were generally higher than what was reported by a study in Nottingham which reported only 6.6% of respondents suggesting information flow as the area for improvement and 4.1% suggesting more detailed interpretation of the screen images (Whynes [26]) as compared to 12.8% and 23.4%, respectively, in our study (Figure 1). This may be due to the fact that Nottingham respondents were more satisfied with the ultrasound services provided and hence did not offer too many suggestions. Generally, the women considered antenatal ultrasound an essential part of antenatal care with most (90.4%) indicating that they would recommend it to other women and cited detection of the fetal position, fetal development, and pregnancy-related complications as their reasons for the recommendation.

### 4.1. Limitation of the Study

Interpretation of questionnaires to nonliterate clients may introduce interviewer bias. Although responses from the open-ended questions were checked and grouped under common themes, certain opinions may have been suppressed.

## 5. Conclusion

The main reasons for the Ghanaian primigravidas to undergo ultrasound imaging were because the doctor or midwife requested for the scan and also to check for fetal abnormalities. The general knowledge on ultrasound use in pregnancy was high but the knowledge of specific congenital fetal abnormalities that can be detected by ultrasound imaging was generally low. Religious considerations did not influence the participants in their decision to undergo the ultrasound scanning, which is encouraging in the Ghanaian/African setting where religion plays a key role in the way things are done. The main expectations of the respondents in this study were to know gender, pregnancy-related complications, and abnormalities in the fetus. The most common suggestions stated by the participants to make their future experiences with ultrasound better were that the practitioners should allow them to view the scan monitor during the procedure, be provided with more accurate ultrasound findings, and the ultrasound examination be made affordable in terms of pricing. The perception of the respondents on ultrasound imaging during pregnancy was largely positive.

### 5.1. Implications for Practice

Expectations from the women to diagnose congenital abnormalities were moderate and hence practitioners must be skilled enough to meet such expectations. Low knowledge about specific fetal abnormalities that ultrasound can help diagnose means practitioners must give more education. Provision of modern equipment to improve detection of more complications and to improve accuracy of findings is encouraged.

## Figures and Tables

**Table 1:** Sample size determination for each site.

Facility	Ultrasound throughput 2018	Number of participants at each site
CCTH	8436	237
Adisadel	3120	88
AGH	2080	59
Total	13636	384

**Table 2:** Sociodemographic characteristics of participants.

Variable	Frequency (%)
Age of respondents	
11–20 years	41 (10.7)
21–30 years	180 (46.9)
31–40 years	148 (38.5)
41–50 years	15 (3.9)
Level of education	
No formal education	21 (5.5)
Basic education (class 1 to junior high)	181 (47.1)
Secondary education	89 (23.2)
Tertiary education	93 (24.2)
Religion	
Christian	336 (87.5)
Muslim	48 (12.5)
Occupation	
Farmer	5 (1.6)
Trader	132 (41.6)
Civil servant	16 (5.0)
Housewife	6 (1.9)
Self-employed	5 (1.6)
Vocational	69 (21.8)
Professionals	84 (26.5)
Marital status	
Single	85 (22.1)
Married	293 (76.3)
Divorced	1 (0.3)
Widowed	1 (0.3)
Separated	4 (1.0)

**Table 3:** Respondents views about obstetric ultrasound scans.

Variable	Frequency (%)
Do you know what ultrasound is used for?	
Yes	337 (87.8)
No	47 (12.2)
What effects do you think ultrasound can have on your pregnancy outcome?	
Positive effects	378 (98.4)
Negative effects	6 (1.6)
Will the cost of ultrasound scanning influence your decision to undergo the examination next time?	
Yes	374 (97.4)
No	10 (2.6)
Why the cost will influence your decision?	
Expensive services	171 (45.7)
Captured under NHIS	84 (22.5)
Free services	119 (31.8)

NHIS: National Health Insurance Scheme.

**Table 4:** The women's reasons for undergoing ultrasound and expectations for the scan.

Variable	Frequency (%)
*Reasons*	
Requested by doctor	
Yes	336 (87.5)
No	48 (12.5)
Relevant to management of pregnancy	
Yes	184 (47.9)
No	200 (52.1)
To estimate date of delivery	
Yes	150 (39.1)
No	234 (60.9)
To check the number of fetuses	
Yes	133 (34.6)
No	251 (65.4)
To check the gender of fetus	
Yes	146 (38.0)
No	238 (62.0)
To check for abnormalities in the fetus	
Yes	207 (53.9)
No	177 (46.1)
A religious leader advised me to do it	
Yes	0 (0.0)
No	384 (100.0)

*Expectations*	
Know gender of fetus	
Yes	224 (58.3)
No	160 (41.7)
See fetus before birth	
Yes	126 (32.8)
No	258 (67.2)
Pregnancy-related complications	
Yes	223 (58.1)
No	161 (41.9)
Know placenta is located normally	
Yes	184 (47.9)
No	200 (52.1)
Know any abnormalities in fetus	
Yes	221 (57.6)
No	163 (42.4)
Know the number of fetuses	
Yes	141 (36.7)
No	243 (63.3)

**Table 5:** The women's reasons for recommending ultrasound scanning to other women.

Variable	Frequency (%)
Will you recommend ultrasound to other women?	
Yes	347 (90.4)
No	37 (9.6)
Reasons for the recommendations	
Detect congenital abnormalities	97 (28.0)
Detect fetal position, development, and pregnancy-related complication	107 (30.8)
Management of pregnancy and planning	86 (24.8)
To know the estimated date of delivery and confirm pregnancy	57 (16.4)

**Table 6:** A cross-tabulation of the levels of education of participants against women's reasons for undergoing ultrasound scanning and knowledge of congenital abnormalities that ultrasound can diagnose.

Variable	Levels of education				*χ* ^2^	*P* value
	No formal education	Basic education	Secondary education	Tertiary education		
*The women's reasons for undergoing ultrasound*						
Requested by doctor						
Yes	15	157	81	83	6.317	0.097
No	6	24	8	10		
Relevant to management of pregnancy						
Yes	8	78	35	63	19.777	**0.000** ^*∗*^
No	13	103	54	30		
Estimate date of delivery						
Yes	5	54	36	55	24.349	**0.000** ^*∗*^
No	16	127	53	38		
Check number of fetuses						
Yes	5	50	30	48	16.891	**0.001** ^*∗*^
No	16	131	59	45		
Check gender of fetus						
Yes	4	61	36	45	9.105	**0.028** ^*∗*^
No	17	120	53	48		
Abnormalities in fetus						
Yes	9	88	51	59	6.884	0.076
No	12	93	38	34		

*Knowledge of congenital abnormalities that ultrasound can diagnose*						
Abnormal head development						
Yes	13	112	63	58	2.282	0.516
No	8	69	26	35		
Cardiac developmental abnormalities						
Yes	9	93	47	52	1.308	0.727
No	12	88	42	41		
Cleft palate						
Yes	7	85	30	29	8.501	**0.037** ^*∗*^
No	14	96	59	64		
Limb abnormalities						
Yes	8	77	35	45	1.813	0.612
No	13	104	54	48		
Fetal organ abnormalities						
Yes	5	45	25	46	18.654	**0.000** ^*∗*^
No	16	136	64	47		
Down's syndrome						
Yes	0	27	13	27	14.407	**0.002** ^*∗*^
No	21	154	76	66		

^*∗*^Statistically significant. Pearson's Chi-square (*χ*^2^) was used to examine the relationship between levels of education of participants against women's reasons for undergoing ultrasound scanning and their knowledge of congenital abnormalities that ultrasound can help diagnose. *P* value ≤0.05 is statistically significant.

## Data Availability

The data used to support the findings of this article may be released upon request to the Head of the Research Unit of the Cape Coast Teaching Hospital. Postal address is as follows: P.O. Box CT I363, Cape Coast, Ghana. E-mail is ccthresearch@gmail.com.
